# Experimental Approach to Evaluate the ^11^C Perfusion and Diffusion in Small Animal Tissues for HadronPET Applications

**DOI:** 10.1371/journal.pone.0151212

**Published:** 2016-03-25

**Authors:** Immaculada Martínez-Rovira, Raphaël Boisgard, Géraldine Pottier, Bertrand Kuhnast, Sébastien Jan

**Affiliations:** 1 IMNC - UMR 8165 CNRS/Univ Paris 11/Univ Paris 7 - Orsay, France; 2 CEA/I2BM/SHFJ - IMIV, UMR 1023 Inserm/CEA/Univ Paris 11, ERL 9218 CNRS - Orsay, France; University of Manchester, UNITED KINGDOM

## Abstract

The development of a reliable dose monitoring system in hadron therapy is essential in order to control the treatment plan delivery. Positron Emission Tomography (PET) is the only method used in clinics nowadays for quality assurance. However, the accuracy of this method is limited by the loss of signal due to the biological washout processes. Up to the moment, very few studies measured the washout processes and there is no database of washout data as a function of the tissue and radioisotope. One of the main difficulties is related to the complexity of such measurements, along with the limited time slots available in hadron therapy facilities. Thus, in this work, we proposed an alternative *in vivo* methodology for the measurement and modeling of the biological washout parameters without any radiative devices. It consists in the implementation of a point-like radioisotope source by direct injection on the tissues of interest and its measurement by means of high-resolution preclinical PET systems. In particular, the washout of ^11^C carbonate radioisotopes was assessed, considering that ^11^C is is the most abundant *β*^+^ emitter produced by carbon beams. ^11^C washout measurements were performed in several tissues of interest (brain, muscle and 9L tumor xenograf) in rodents (Wistar rat). Results show that the methodology presented is sensitive to the washout variations depending on the selected tissue. Finally, a first qualitative correlation between ^11^C tumor washout properties and tumor metabolism (via ^18^F-FDG tracer uptake) was found.

## Introduction

Hadron therapy consists in the use of accelerated proton and ions (mainly carbon) for cancer treatment. The main advantages of hadron therapy with respect to conventional radiotherapy are the selective energy dose deposition in depth and the increased biological effectiveness around the Bragg peak. The combination of these properties results in a significant destructive effect on the tumor, while sparing the surrounding healthy tissue [1–3]. The development of a reliable dose monitoring system in hadron therapy is essential in order to control the treatment plan delivery. Positron Emission Tomography (PET) devices are clinically used for quality assurance to scan the secondary *β*^+^ emitters (such as ^11^C and ^15^O, among others) produced during nuclear reactions. It has been proven that the use of PET imaging for dose monitoring enables control of the dose falloff position and thus, detection of deviations between the planned and delivered dose distributions [4, 5]. However, the accuracy of this method is limited by the loss of signal due to the biological washout [6, 7]. The study of these processes is essential to reproduce the measured PET activity distributions, since they can significantly alter the spatial distribution of the signal and the quantification associated to the target volume (see [8–11], among others). Previous Monte Carlo studies have quantified the effect of the washout processes in carbon therapy treatments using a full Monte Carlo simulation approach [11]. Results showed that the intrinsic biological variability in a tumor in terms of its vascularization has a strong impact on the final PET activity distributions. Thus, for a given tumor dose prescription, there is an important variability in the PET activity distributions. The activity profiles showed significant differences in the estimation of the distal activity falloff, in addition to fluctuations of a factor 2 in the absolute activity between active and hypoxic tumor models. This means that it is difficult to provide an absolute dose quantification if no information on the tumor perfusion status is considered [11]. The main challenge of these type of measurements is related to the experimental estimation of the tumor washout parameters in the way to quantify the dose delivery based on the PET acquisition analysis. To our knowledge, very few studies attempted to measure the washout processes [6, 7, 12, 13]. Moreover, there is no comprehensive database of washout data as a function of the tissue and radioisotope. One of the main difficulties is related to the complexity of such measurements, along with the limited time slots in hadron therapy facilities. Thus, in this work, we propose an alternative *in vivo* methodology for the measurement and modeling of the biological washout parameters without any radiative devices. The main idea consists in the direct injection of a ^11^C radioisotope point-like source in the tissues of interest (a rodent model for this validation study). We focused on ^11^C since it is the most abundant *β*^+^ emitter produced by ^12^C in water [14]. A dynamic PET acquisition using a high resolution pre-clinical system was simultaneously started with the injection to assess time-activity distributions. The first objective of this study was to assess the potential of this approach in the characterization and discrimination of biological washout parameters. The second objective consisted in the evaluation of the potential correlation between ^11^C washout properties and tumor metabolism. This was done by quantifying the glucose tumor consumption using ^18^F-FDG, which is the most used tracer in clinical application for diagnosis. The idea of these last measurements was to have a first hint for potential future clinical applications.

## Materials and Methods

### The washout model

Mizuno and collaborators measured the washout processes for dose monitoring in hadron therapy [6, 7]. For this purpose, radioactive ^12^C beams were impinged on rabbit brain and tight muscle. Time-activity distributions were recorded by using a trivial PET scanner based on a pair of Anger-type scintillation camera [15] in alive and death conditions. By comparing the two sets of experimental data, the washout processes were modeled. Measured activity distributions [*A*_meas_(t)] were divided into two parts: a biological component [*C*_bio_(*t*)] related to the washout, blood perfusion and tissue diffusion effects; and a physical activity part [*A*_phys_(*t*)], which is induced by the isotope radioactive decay time. Thus, the PET activity distributions were expressed as follows:

$$A_{\text{meas}}\left(t\right)=C_{\text{bio}}\left(t\right).A_{\text{phys}}\left(t\right)$$

In the Mizuno *et al.* approach, the biological component [*C*_bio_(*t*)] was modeled by adding three exponential components:

$$C_{\text{bio}}\left(t\right)=M_{\text{biof}}.e^{-\lambda_{\text{biof}}.t}+M_{\text{biom}}.e^{-\lambda_{\text{biom}}.t}+M_{\text{bios}}.e^{-\lambda_{\text{bios}}.t}$$

where *λ*_biof_, *λ*_biom_ and *λ*_bios_ represent the decay constants, and *M*_biof_, *M*_biom_ and *M*_bios_ are the fractions (*M*_biof_+*M*_biom_+*M*_bios_ = 1) of the fast, medium and slow components. Regarding the biological interpretation of these components, the fast one is related to the blood flow and occurs during the first seconds; the medium one is due to the micro-circulation and has a half-life of several minutes; and the slow component consists in the trapping of the radioisotopes by the stable molecules of the organ, which are metabolized only slowly (hours).

Our proposed model is based on the Mizuno approach, including a few modifications. The measured activity [*A*_meas_(t)] is only expressed in terms of a biological component [*C*_bio_(t)], since activity distributions were previously corrected by physical radioactive decay. At the same time, the biological component is divided into two components (tissular and washout).

$$A_{\text{meas}}\left(t\right)=A_{\text{bio}}\left(t\right)=A_{0}.C_{\text{bio}}\left(t\right)=A_{0}.C_{\text{tiss}}\left(t\right).C_{\text{wash}}\left(t\right)$$

The tissular component [*C*_tiss_(*t*)] takes into account the passive diffusion tissue characteristics, while the washout component [*C*_wash_(*t*)] models blood perfusion effects. It is important to mention that the tissular component was not directly considered by Mizuno and collaborators.

Equivalent PET acquisitions were performed under alive [*C*_bio,alive_(*t*)] and dead [*C*_bio,dead_(*t*)] conditions to evaluate the two components of the model. In this study, both components were modelled by a single exponential decay. Despite Mizuno *et al.* modelled the washout by using three additive exponential components, posterior studies showed that the biological (washout) part could be modelled by a single exponential component [13], as proposed in this work.

In alive conditions, PET activity distributions contain both components (tissular and washout) as follows:

$$C_{\text{bio},\text{alive}}\left(t\right)=C_{\text{tiss}}\left(t\right).C_{\text{wash}}\left(t\right)=\exp^{-\lambda_{\text{wash}}.t}.\exp^{-\lambda_{\text{tiss}}.t}$$

The tissular diffusion component can be evaluated under dead conditions, since there is no tissue perfusion (washout) for these measurement conditions.

$$C_{\text{bio},\text{dead}}\left(t\right)=C_{\text{tiss}}\left(t\right)=e^{-\lambda_{\text{tiss}}.t}$$

These two acquisitions allow to discriminate between different tissue properties depending on the ^11^C isotope circulation.

### Experimental set-up

#### Animal model and ^11^C compound injection

The Wistar rat model was used for this study (males around 300 g, 7–9 weeks old). The selected tissues of interest were: brain (striatum), leg muscle and xenograft tumor obtained by subcutaneous injection of 9L glioma cells. A group of *N* = 4 animals was used for each type of tissue. The ^11^C solution was directly injected in the tissues of interest using a Hamilton syringe on anesthetized rat with isoflurane. To facilitate the injection on muscle or tumor, the skin was previously incised. For brain injection, scalp was incised and skull was perfored using drill at precise coordinate (RC +0.7, ML -2.7 from bregma) in order to give access to the brain in the striatal area. The needle of the hamilton syringe was introduced from 5.4 mm (DV) from the brain surface. To be in a situation of a ^11^C point-like-source, the injected volume was calibrated at 1 *μ*L and the total injected activity was in the range between 0.7 and 1.1 MBq.

Animals were anesthetized during the PET acquisition by using isoflurane to evaluate the vascular component. Just before the second PET scan (2–3 hours later), animals were euthanasied with an intravenous injection of pentobarbital (180 mg/kg) for the measurement of the tissular diffusion component. This study was carried out in strict accordance with the recommendations in the Guide for the Care and Use of Laboratory Animals of the National Institutes of Health. The protocol was approved by the Committee on the Ethics of Animal Experiments of the CEA (Permit Number: 47).

#### ^18^F-FDG activity versus ^11^C washout parameters

In order to evaluate a potential correlation between the tumor washout parameter values and glucose metabolism activity, PET acquisition with ^18^F-FDG intravenous injections were performed on rats with xenograft tumors. Subsequently, ^11^C perfusion measurements were made to verify if it was possible to predict ^11^C infusion rates based on the physiological state of the tumor.

### Chemistry of ^11^C compound

As it is not possible to inject a pure ^11^C solution, ^11^C-labeled carbonate anions were injected. This corresponds to a possible fate of ^11^C ions induced by hadron therapy beams [16]. [^11^C]CO_2_ was produced via the ^14^N(p,*α*)^11^C nuclear reaction by irradiation of a N_2_/O_2_ target mixture (99.5/0.5 ultrapure, Air Liquide) with an 18 MeV proton beam (at 25 ÂμA) on an IBA Cyclone-18/9 cyclotron. At the end of irradiation (EOB), [^11^C]CO_2_ is automatically transferred to the TRACERLab FX C Pro (General Electric Medical Systems) synthesizer placed in a ventilated, 5 cm-lead shielded hot cell. The radioactive gas is trapped at room temperature on Shimalite-Ni sieve. After completion of the transfer from the cyclotron, the trap is heated to 380Â°C and [^11^C]CO_2_ is released under a flow of helium (10 mL/min), transferred to an adjacent 5 cm-lead shielded hot cell through a TeflonÂ^®^ line, and finally bubbles for 10 min in a 1N aq. NaOH solution (5 mL) at room temperature to form [^11^C]carbonate ([^11^C]CO_3_^2−^, Na^+^). The sodium hydroxide solution was placed in a shielded vial equipped with a needle exhaust connected to a SodalimeÂ^®^ trap prior to [^11^C]CO_2_ transfer. Typically 18.5 to 22.0 GBq of [^11^C]carbonate can be produced in 15 minutes starting from a 37 GBq [^11^C]CO_2_ batch.

### PET acquisition and data analysis

For this study, the PET scanner system used was a SIEMENS INVEON dedicated to pre-clinical studies. The spatial resolution of this system is around 1.5 mm at the center of the field of view (FOV) and the sensitivity is close to 10% for a point-like-source placed at the center of the FOV. The protocol was defined with an acquisition time of 30–60 min. The listmode file was then histogramed to have a set of 25 frames with the following dynamic sequence: 10×30 s, 5×60 s and 10×300 s. Data was normalized, corrected by attenuation, scattering, arc effects and physical decay. Finally, images are reconstructed by using a Fourier Rebinning algorithm and OSEM-2D iterative method.

To perform the analysis, a region of interest (ROI) has been manually selected containing the pixels with activity values higher than 50% of the maximum activity in the first frame. Then, masks were applied to the rest of frames to have access to the complete time-activity curves (TACs). By comparing the two sets of experimental data (dead and alive), the washout processes were modeled. The half-life value of the tissular component (*T*_1/2,tiss_ = ln2/*λ*_tiss_) was obtained from the time-activity distributions in the dead measurements. Instead, the half-life of the washout component (*T*_1/2,wash_ = ln2/*λ*_wash_) was evaluated from the activity distributions in the alive animals. See more details of the model in ‘The washout model’ section.

### Activity distributions

**Fig 1:**
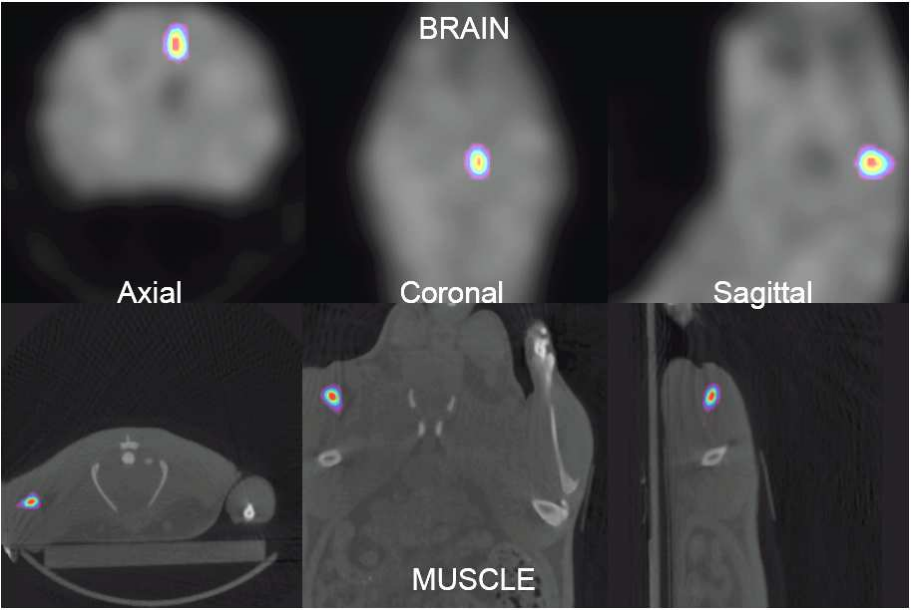
Axial view of the ^11^C activity distributions at the beginning of PET acquisition (in the brain at the top, and in the muscle at the bottom). PET images were fused with X ray CT scan for a better injected point localization.

Fig 1 illustrates the axial view of the reconstructed images just after the injection in the tissues of interest (brain at the top, and leg muscle at the bottom). It can be seen that ^11^C distributions are represented by a point-like-source. The point spread function of the activity distributions is around 2–3 mm (Full Width at Half Maximum, FWHM).

### Diffusion or tissular component

In this section, results obtained from the dead animal experiments are presented. We made the hypothesis that the diffusion of the tracer from the injection point is mainly driven by the concentration gradient. This phenomenon can be estimated in non-perfused tissues. In this way, the tissular component and the corresponding half-life (*T*_1/2,tiss_) can be extracted from these measurements.

**Fig 2:**
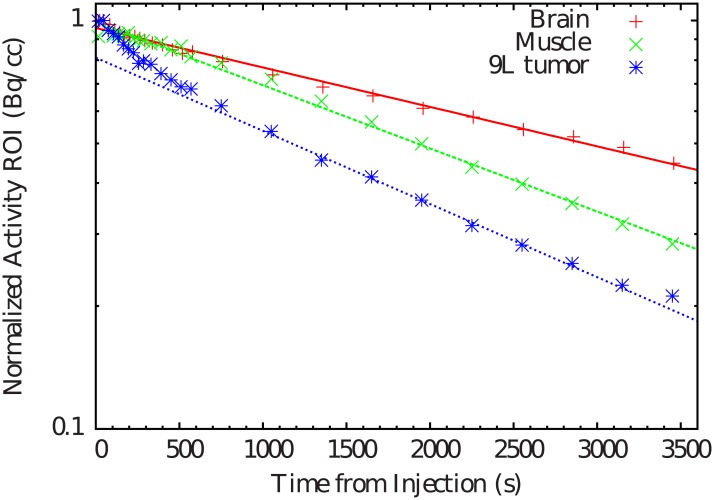
Time-activity curves of ^11^C induced by the diffusion or tissular component in the brain, leg muscle and tumor.

Fig 2 represents the time-activity curves. As expected, activity is decreasing as a function of time due to the passive diffusion tissue characteristics. In the case of the tumor, there is a few change of trend at 500 s after starting the acquisition. This could be explain by the re-equilibration of the pressure in the tumor since *in vivo* tumor tissue pressure is higher than in normal tissues. The time needed to re-equilibrate the pressure after sacrifice could explain the variation in the ^11^C tumor kinetics observed in the curve.

Data corresponding to different tissues are well-differentiated (brain, muscle and tumor). Table 1 shows the average *T*_1/2,tiss_ values, which are in the order of 30–40 min. Passive diffusion is faster in the tumor in comparison to normal organs.

**Table 1:** ^11^C *T*_1/2,tiss_ (and the corresponding standard deviation values) of the diffusion or tissular component in the brain, leg muscle and tumor.

	Brain	Muscle	Tumor
*T*_1/2,wash_(*s*)	2878±155	2132±230	2019±188

### Vascular or washout component

**Fig 3:**
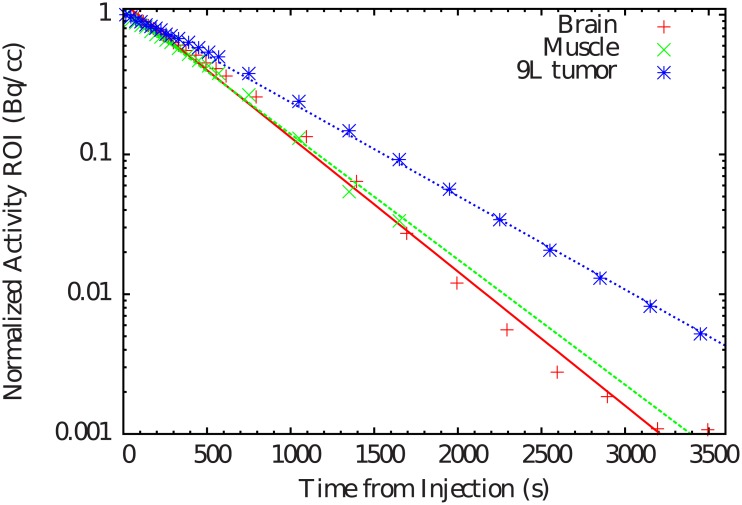
Time-activity curves of ^11^C induced by the vascular or washout component in the brain, leg muscle and tumor.

The decay of the biological component is the combination between passive diffusion estimated previously and active transport from the point of injection due to fluid movement such as lymph or blood circulation present in living animals. In this section, results obtained from the alive animal experiments are presented. Time-activity curves are represented in Fig 3, with a decreasing trend as a function of time. The corresponding *T*_1/2,wash_ values are listed in Table 2 and they are in the order of several minutes. No significant differences are observed between brain and muscle, as reported in previous works [6, 13]. *T*_1/2,wash_ values for tumor are clearly larger than in the case of brain or muscle, illustrating a larger half-life, corresponding to a lower washout.

**Table 2:** ^11^C *T*_1/2,wash_ (and the corresponding standard deviation values) of the vascular or washout component in the brain, leg muscle and tumor.

	Brain	Muscle	Tumor
*T*_1/2,tiss_(*s*)	330±51	361±186	621±151

### Glucose metabolism versus washout parameters

**Fig 4:**
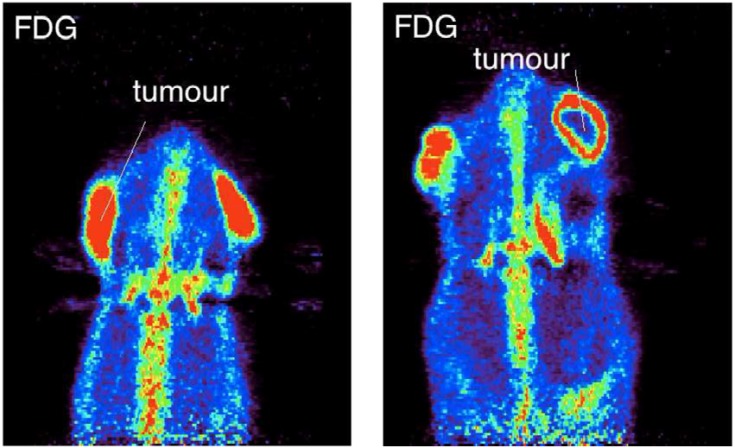
^18^F-FDG distribution in case of active tumor (left) and necrotic or hypoxic tumor (right).

**Fig 5:**
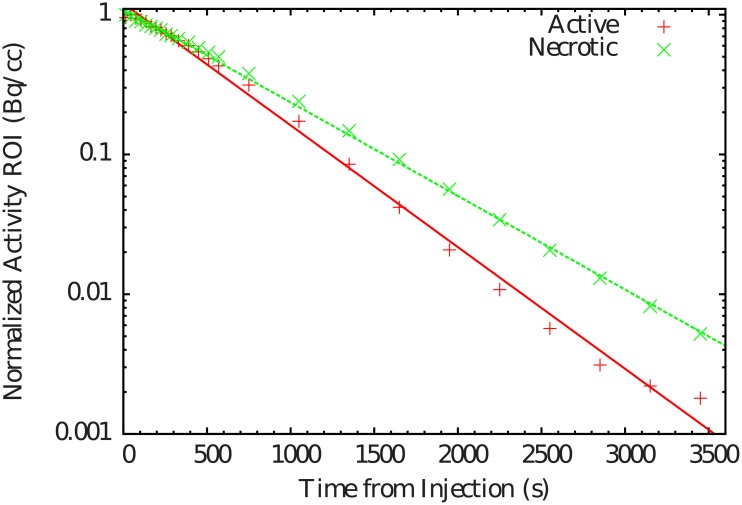
Time-activity curves of ^11^C induced by the vascular or washout component in different tumor metabolic states (necrotic and active tumors).

The objective of this part consisted in the evaluation of the potential correlation between the ^11^C washout properties and tumor metabolism. Fig 4 represents PET images of the ^18^F-FDG distributions in rats, showing different uptakes in xenograpf tumors for different tumor metabolic states. The animal on the right of the figure display a necrotic tumor caracterised by the lack of ^18^F-FDG uptake in the center of the tumor. It is well known that ^18^F-FDG uptake is directly correlated to the tumor biological activity. The corresponding time-activity curves and *T*_1/2,wash_ values are shown in Fig 5 and Table 3, respectively. It is clearly seen that *T*_1/2,wash_ are different depending on the tumor metabolic state. Smaller *T*_1/2,wash_ values are observed for more active tumors, illustrating a higher half-life of the tracer.

**Table 3:** ^11^C *T*_1/2,wash_ values of the vascular or washout component in different tumor metabolic states (necrotic and active tumors).

	Necrotic tumor	Active tumor
*T*_1/2,wash_(*s*)	613	418

## Discussion

The first topic that need to be discussed is related to the specificity of the tracer. Hydrogenocarbonate anions (HCO_3_^−^) are present in all body fluids and diffuse in all organs. It is a major component of the pH buffering system of the human body mediated by the equilibrium between carbonic acid (CO_2_) and hydrogenocarbonates (HCO_3_^−^). Infused as [^11^C]CO_2_ or injected as [^11^C]CO_3_^2−^, these entities are immediately buffered as [^11^C]HCO_3_^−^ that diffuse with fluids. Radioactivity is rapidly distributed in organs and released as [^11^C]CO_2_. It has been demonstrated that less than 20% of radioactivity was recovered in the blood as entities different from [^11^C]CO_2_ or [^11^C]HCO_3_^−^ at 60 min after injection in dogs [17]. Hydrogenocarbonate anions represent thus an interesting model of diffusion and elimination of water-soluble simple carbon-containing entities that could be generated by carbon beams. The slope variability in the tumoral diffusion or tissular component is another point of discussion. The enhanced permeability and retention (EPR) effect in solid tumors is driven by abnormalities in the tumor microenvironment, including heterogeneity in vascular permeability and elevated interstitial fluid pressure (IFP). This well-known biological effect is largely used to develop therapeutic strategies in oncology. This increase of IFP have been explained by the lack of lymphatic drainage vessels inside the tumor in comparison to the blood influx. This phenomenon is more important in larger tumors than in smaller ones, and could be described by a gradient increasing from the periphery of the tumor to its center. In our study, intratumoral injection of soluble ions (such as carbonate) presents less diffusion from the injection point than those observed for normal tissues (*i.e.*, brain and muscle). This observation can be explained by the retention effect limiting tumoral efflux of fluids and ions. In case of euthanized animals, the tumoral equilibrium between influx (mainly from blood) and efflux (mainly from lymphatic vessels) is abolished. This gradient disruption is accompanied by a rapid pressure equilibrium inducing a “wave” of water and soluble molecules at the opposite of initial pressure gradient. This mechanism certainly explains the observation of the accelerated reduction of carbonate observed in tumor in comparison to the other tissues (brain and muscle). The high lipid content in brain in comparison to muscle can also explain the difference of diffusion measured between these two tissues. Regarding the washout component, we observed that the methodology used here is sensitive to the washout as a function of tissue (tumor *versus* brain/muscle). In agreement with previous works, no significant differences are detected between brain and muscle [6, 13]. Finally, results presented in the last section show a first qualitative proof of concept about the correlation between ^11^C washout properties and tumor metabolism. However, in order to be able to have a quantitative model to extract washout parameters from clinical PET data, it will be clearly necessary to define a systematic study including a significant statistical animal group. It would be also necessary to define a specific co-registration between the ^18^F-FDG and the ^11^C images to extract the correlation parameters with voxel by voxel analysis. This part is out of the scope of our study and will be evaluated in a future work.

## Conclusions

Washout processes play a major role in PET dose monitoring in hadron therapy. In this work, a new methodology for the washout measurement is proposed. Results show that biological washout parameters can be evaluated and studied by using a dynamic PET scan associated to a point-like-source injection of a positron emitter within the organ targets of a rodent model. The methodology presented is sensitive to the washout variations depending on the selected tissue.

In addition, a qualitative correlation has been found between the perfusion and diffusion of ^11^C isotopes within the tumor and the tumor tracer uptake (^18^F-FDG in our case). This gives a first hint for a next quantitative study including a statistical significant animal pool.

Next steps include the association of the washout measurements with functional characterization of tissues, *i.e.*, using tracers for metabolism or hypoxia (such as ^18^F-FMISO or others). The main idea is to obtain a relation between the tumor heterogeneity (estimated by PET) and the impact on the biological washout parameters.
